# Antidiabetic Potential of *Prosopis farcta* Roots: In Vitro Pancreatic Beta Cell Protection, Enhancement of Glucose Consumption, and Bioassay-Guided Fractionation

**DOI:** 10.1155/2020/8048273

**Published:** 2020-01-20

**Authors:** Behzad Shahbazi, Saba Feyzmand, Fataneh Jafari, Nastaran Ghiasvand, Gholamreza Bahrami, Ali Fattahi, Solomon Habtemariam, Seyed-Mohammad Nabavi, Yalda Shokoohinia

**Affiliations:** ^1^Pharmaceutical Sciences Research Center, Health Institute, Kermanshah University of Medical Sciences, Kermanshah, Iran; ^2^Student Research Committee, School of Pharmacy, Kermanshah University of Medical Sciences, Kermanshah, Iran; ^3^Medical Biology Research Center, Health Technology Institute, Kermanshah University of Medical Sciences, Kermanshah, Iran; ^4^Pharmacognosy Research Laboratories & Herbal Analysis Services UK, University of Greenwich, Central Avenue, Chatham-Maritime, Kent ME4 4TB, Gillingham, UK; ^5^Applied Biotechnology Research Center, Baqiyatallah University of Medical Sciences, Tehran 14359-16471, Iran; ^6^Ric Scalzo Research Center, Southwest College of Naturopathic Medicine, Tempe, AZ, USA

## Abstract

By using the streptozotocin- (STZ-) induced cytotoxicity in *β*-TC3 cells as an assay model, a bioassay-guided fractionation study was employed to isolate and characterize the potential antidiabetic principles of roots of *Prosopis farcta.* A combination of open column chromatography on reverse-phase silica gel using a water-ethanol gradient (10 : 90 to 100 : 0) followed by HPLC-based fractionation led to an active compound that appears to be composed of carbohydrate/sugar. When cell viability under STZ was reduced to 49.8 ± 4% (mean ± SD), treatment with the active compound at the concentration of 0.5 mg/mL either as a coadministration or a pretreatment improved the viability to 93 ± 1.9% and 91.5 ± 7%, respectively. The reduction in the mitochondrial membrane potential by STZ (47.34 ± 8.9% of control) was similarly recovered to 84.5 ± 4.3 (coadministration) and 88 ± 5.5% (pretreatment) by the active fraction. The bioassay-guided fractionation, *β*-cell protective effect, and increased glucose consumption (up to 1.49-fold increase) in hepatocytes by the extracts and active fraction are also discussed.

## 1. Introduction

Type 2 diabetes (T2D) is a metabolic disorder characterized by insulin resistance which may also be combined with insufficient amount of insulin release from pancreatic *β*-cells [1]. In addition to genetic factors [2], obesity and lack of physical activity are well-established risk factors for T2D [3]. The mortality and morbidity of patients with T2D are associated with the micro- (retinopathy, nephropathy, and neuropathy) and macrovascular (ischemic heart disease, peripheral vascular disease, and cerebrovascular diseases) complications associated with hyperglycemia [4, 5]. While antidiabetic drugs in current use (e.g., sulphonylureas such as glibenclamide) offer some benefits, they also induce undesirable side effects such as hypoglycemia, weight gain, skin reactions, acute porphyria, and rarely hyponatremia [5]. The use of plants for the treatment of diabetes goes back to more than 3,500 years, with many of them considered to be associated with relatively little side effects compared with synthetic drugs [6]. Hence, the search for novel natural product-based therapeutic agents for complex metabolic disorders is currently advocated [7, 8].

*Prosopis farcta* (family Fabaceae) is a well-known medicinal plant that grows in Jordan, Kuwait, Turkey, Iraq, Northern Africa, South Western Asia, and Iran (Khuzestan, Gilan, Fars, Hormozgan, Baluchestan, Khorasan, and Tehran provinces). The plant commonly known by its local name as the “Syrian mesquite” generally grows in warm and dryer climate [9].

In traditional medicine, *P. farcta* is known to be used for treating various disease conditions including neurological disorders [10], hyperlipidemia [11], diabetes, inflammatory diseases [12], wounds and skin disorder, prostate disorders [13], measles, urinary [14], cardiac or chest pain, angina pectoris [15], infectious disease, diarrhea, colds [13], and hepatic diseases [4]. An indication on the antidiabetic potential of the roots has also been outlined in our previous preliminary studies [16]. In the current study, we employed in vitro studies to fractionate the crude extract and attempted to isolate the active principle. The effect of the carbohydrate/sugar components as active principle to ameliorate the streptozotocin- (STZ-) induced cytotoxicity in pancreatic *β*-cells (*β*-TC3) and increased glucose consumption in hepatocytes are discussed.

## 2. Materials and Methods

### 2.1. General Instruments and Chemicals

^1^H NMR spectra were measured on a Bruker DRX-500 AVANCE instrument. Chemical shifts were referenced to the residual solvent signal (CD_3_OD : D_2_O; *δ*_H_ 3.3, 4.8). Separations were monitored, and fractions were pooled by TLC on Merck 60 F254 (0.25 mm) plates (butanol : acetic acid : H_2_O; 4 : 1 : 5) and were visualized by UV inspection and/or staining with Ce(SO_4_)_2_/molybdate and heating. HPLC purifications were achieved on a Young Lin apparatus equipped with a binary pump (YL 9111S) and photodiode array detector (YL 9160). Vertica (reverse phase, RP18 250 × 30 mm) columns were used, with 10 mL/min as flow rate. The cell viability and mitochondrial membrane potential were measured by a plat reader (Hybrid SynergyH1, Biotec, USA).

Cell culture plates were products of NEST (China). Glucose assay kit (reagents including glucose oxidase/peroxidase (G-3660), *O*-dianisidine reagent (D-2679), glucose standard solution (G-3285)), 3-(4,5-dimethylthiazol-2-yl)-2,5-diphenyltetrazolium (MTT), dimethyl sulfoxide (DMSO), rhodamine 123, Triton X-100, and streptozotocin (STZ) were purchased from Sigma-Aldrich, USA. Dulbecco modified Eagle medium (DMEM), fetal bovine serum (FBS), bovine serum albumin (BSA), penicillin-streptomycin, and trypsin were purchased from Atocell, Hungary. All the solvents used for extraction and/or fractionation were obtained from Merck Chemical Company (Darmstadt, Germany).

### 2.2. Plant Material

The roots of *P. farcta* were collected from western Ilam surroundings, Iran, at *ca.* 1400 m above sea level. The plant material was identified by Dr. Shahram Miraghayi at the Medical Biology Research Center of KUMS (Kermanshah, Iran), and the voucher specimen (No. 1002 RUH) was deposited at the Herbarium of Agricultural Faculty of Razi University, Kermanshah, Iran.

### 2.3. Extraction of the Plant Material

The air-dried plant material was subjected to hot water extraction. Briefly, 3.2 L of boiling water was added to 400 g of the root powder in a flask and left for 30 min on a water bath at 90°C. The extract was then filtered and concentrated under reduced pressure using a rotary evaporator to yield 36.92 g of the crude extract. The crude extract was then kept at −20°C until used.

### 2.4. Fractionation of the Crude Extract Using Open Column Chromatography

The crude extract (36.92 g) was fractionated with vacuum liquid chromatography over RP18 and eluted with a H_2_O : EtOH gradient (90 : 10 to 0 : 100). Seven fractions were collected (F1–F7). A portion (3.35 g) of fraction 2 (F2) that showed antidiabetic activity in the in vitro STZ-induced *β*-TC3 cell assay was further fractionated on RP18 using H_2_O : MeOH (95 : 5 to 50 : 50) to yield 4 subfractions, F2A–F2D.

### 2.5. High-Performance Liquid Chromatography Purification of Active Subfractions

**Scheme 1:**
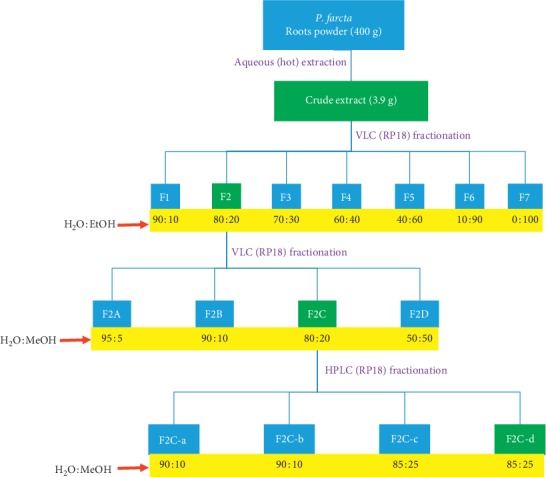
Overview of the bioassay-guided fractionation of aqueous extract of *P. farcta* roots. The crude extract was subjected to two steps of vacuum liquid chromatography (VLC) and HPLC fractionations to yield the active fraction (F2C-d) that showed effect on pancreatic *β*-cells and hepatocytes in vitro. Note the active fractions in the green boxes as F2, F2C, and F2C-D.

The subfraction F2C was purified using Vertica (reversed phase, RP18 250 × 30 mm) column using a gradient of H_2_O : MeOH from 90 : 10 to 85 : 25 to afford four fractions, F2C-A to F2C-D. The fractions were combined on the basis of UV absorption at 210 nm. The most active fraction on the *β*-TC3 cell assay system was F2C-D and was analyzed by ^1^H NMR spectroscopy. The overall fractionation procedure is depicted in Scheme 1.

### 2.6. Cell Culture Conditions

The mouse pancreatic *β*-cell (*β*-TC_3_) and human liver hepatocellular carcinoma (HepG2 Cells) cell lines were purchased from the Iran genetic resources center (Tehran, Iran). Cell cultures were maintained in DMEM medium supplemented with 10% v/v FBS and penicillin/streptomycin (100 U/mL, 100 mg/mL) and kept at 37°C in a humidified atmosphere and 5% CO_2_. The growth medium was changed every 2-3 days and subcultured using trypsin.

### 2.7. Cell Viability Assay

The cytotoxic effect of the crude extract and fractions on *β*-TC_3_ cell line was determined by the MTT colorimetric assay. Cells were plated onto a 96-well plate at a density of 5.0 × 10^4^ cells/mL and in a volume of 180 *μ*L. After the culture was left to establish for 24 h, test samples prepared in water were added in 20 *μ*L volume of various concentrations. The end-point cell viability measurement was based using the MTT assay whereby 20 *μ*L of the stock (5 mg/mL) was added during the last 3 h of the 24 h drug treatment. The medium was then removed by aspiration; the reduced MTT dye in each well was solubilized with 150 *μ*L DMSO, and the absorbance was determined using an ELISA plate reader (synergy H1, Biotek, USA) with a test wavelength of 540 nm and a reference wavelength of 630 [14]. The percentage of viable cells was calculated as follows:

(1) $$\begin{array}{c} \text{percent viability}=\left(\frac{\text{absorbance of test sample}}{\text{absorbance of untreated control}}\right)\times 100. \end{array}$$

### 2.8. Inhibition of STZ-Induced *β*-TC3 Cell Death

The *β*-cell protective effect of the plant samples was assessed using 48 *μ*g/*μ*L of STZ that represent its IC_50_ concentration for lethality in this cell line. The noncytotoxic concentrations of test samples were added either 6 h before or together (coadministration) and cell viability measured using the MTT assay as described above [16].

### 2.9. Glucose Consumption Assay

HepG2 cells maintained in DMEM (5.5 mmol/L glucose plus 10% FBS) were plated onto a 96-well plate and left to establish as confluence over 48 h of incubation. The medium was then replaced by 0.2% BSA and high glucose (11.1 mM) for further 12 h of incubation. After 24 h of incubation with test samples in replicates, culture medium was collected and glucose concentration was measured using an ELISA plate reader (synergy H1, Biotek, USA) with a test wavelength of 540 nm following the instruction of the glucose oxidase kit (Sigma). Glucose consumption was evaluated by measuring the change in absorbance (Δ*A*540) as follows [17]:

(2) $$\begin{array}{c} \text{amount of glucose}\left(\text{mg}\right)\;=\;\frac{\left(\Delta A540\text{ of test}\right)\left(\text{mg glucose in standard}\right)}{\Delta A540\text{of standard}}\;=\frac{\left(\Delta A540\text{ of test}\right)\left(0.05\right)}{\Delta A540\text{ of standard}}. \end{array}$$

### 2.10. Measurement of Mitochondrial Membrane Potential

*β*-TC_3_ cells were seeded in 24-well plates at a density of 10^5^ cells/mL and left to establish for 24 h. After treatment with test samples for 24 h, rhodamine 123 was added for 30 min at 37°C. Cells were then washed three times with phosphate-buffered saline (PBS) and lysed on ice using lysis buffer (1% Triton X-100 in PBS, for 1 h). The fluorescence was measured at an excitation wavelength of 488 nm and emission wavelength of 520 nm using the microplate reader. The mean fluorescence intensity was normalized to the amount of protein present in the sample using Bradford assay [18].

### 2.11. Statistical Analyses

All tests were performed at least in triplicate, and the results were presented as mean ± standard deviation (SD). Analysis of variance (ANOVA) with post hoc Tukey was used to recognize significant differences between the means of the experimental groups using GraphPad Software version 5.0 (GraphPad Software Inc., San Diego, CA, USA). Statistical significance was set at *p* < 0.05.

## 3. Results

### 3.1. Determination of Nontoxic Concentration of Fractions on *β*-TC3 Cell Line

The crude extract of the roots of *P. farcta* was fractioned as described in the experimental section, and the overall approach is depicted in Scheme 1. As shown in Table 1, the fractions (F1-F2) obtained from the crude extract did not display significant cytotoxicity to *β*-TC3 cells at all concentrations tested (0.01–0.5 mg/mL). The subfractions F2A-F2D and final HPLC fractions (F2C-a - 2C-d) (Table 1) did not also display cytotoxicity at the same concentration range. Hence, the chosen concentration range of 0.01–0.5 mg/ml with no sign of toxicity was safe to be used in antidiabetic assay in the *β*-TC3 assay system.

### 3.2. Effect of Different Fractions on STZ-Induced Cytotoxicity in *β*-TC3 Cells

**Figure 1:**
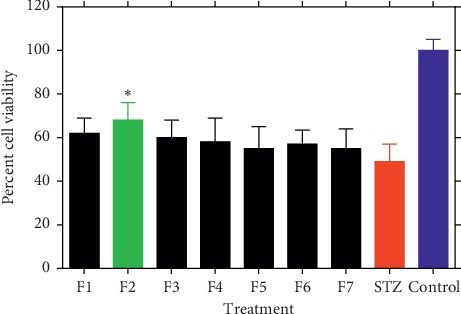
The effect of infusion fraction (F1–F7 at 0.5 mg/mL) coadministration on STZ-induced cytotoxicity in *β*-TC_3_ cells. The cell viability after 24 h of treatment was determined by the MTT assay. Data are expressed as the mean ± SD of three separate experiments. Statistical significance versus the STZ group was calculated by ANOVA (^*∗*^*p* ≤ 0.05).

**Figure 2:**
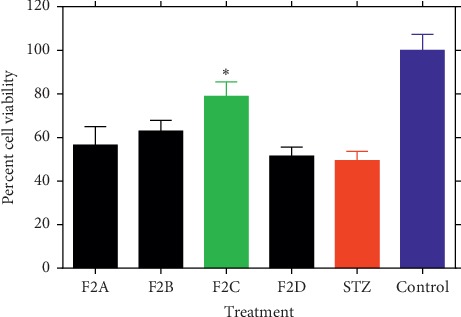
The effect of subfractions of F2A, F2B, F2C, and F2D coadministration (at 0.5 mg/mL) on STZ-induced cytotoxicity in *β*-TC_3_ cells. The cell viability after 24 h of treatment was determined by the MTT assay. Data are expressed as the mean ± SD of three separate experiments. Statistical significance verses the STZ group was calculated by ANOVA (^*∗*^*p* ≤ 0.05).

**Figure 3:**
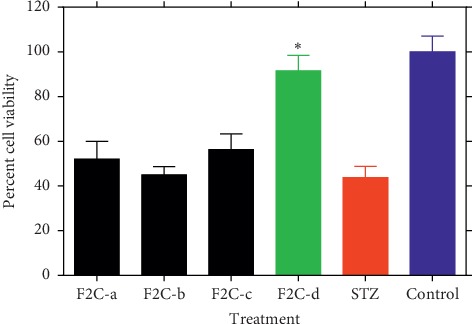
The effect of subfractions of F2C-a, F2C-b, F2C-c, and F2C-d coadministration (at 0.5 mg/mL) on STZ-induced cytotoxicity in *β*-TC_3_ cells. Cell viability after 24 h of treatment was determined by the MTT assay. Data are expressed as the mean ± SD of three separate experiments. Statistical significance versus the STZ group was calculated by ANOVA (^*∗*^*p* ≤ 0.05).

When fractions F1–F7 were screened at the highest nontoxic concentration used (0.5 mg/mL coadministered with STZ) [16], only F2 showed significant protective effect to *β*-TC_3_ cells against STZ (Figure 1). Fraction F2 was therefore selected for further bioassay-guided fraction procedure using reverse-phase silica gel open column chromatography to yield four subfractions (F2A-F2D). Of these, only F2C showed significant cytoprotective effect against STZ (Figure 2). The protective efficiency of F2C was also higher than F2 (approximately 10% higher), suggesting increased bioactivity as the purification proceeds. On this basis, F2C was further fractionated to four subfractions leading to the identification of F2C-d as the active principle (Figure 3). The viability of *β*-TC_3_ cells treated with F2C-d reached close to 100%, i.e., the cytotoxicity of STZ in pancreatic *β*-cells could be completely abolished by F2C-d (Figure 3).

**Figure 4:**
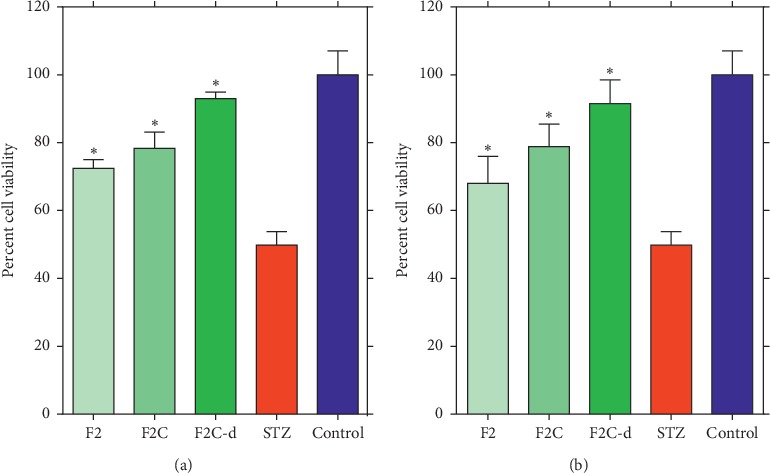
The effect of coadministration (a) and pretreatment (b) with 0.5 mg/mL of F2, F2C, and F2C-d on STZ-induced cytotoxicity in *β*-TC_3_ cells. The viability of cells after 24 h was determined by the MTT assay. Data are expressed as the mean ± SD of three separate experiments. Statistical significance versus the STZ group was calculated by ANOVA (^*∗*^*p* ≤ 0.05).

In the pretreatment protocol where test samples were added 6 h before STZ, the comparative cytoprotective effect of active fractions was also assessed. As shown in Figure 4, cytoprotection were demonstrated for F2, F2C, and F2C-d with the latter being the most potent. No significant difference between pretreatment and coadministration was also observed suggesting the quick onset of action of the active principle(s).

### 3.3. Effects of F2, F2C, and F2C-d Fractions on Mitochondrial Membrane Potential (MMP)

**Figure 5:**
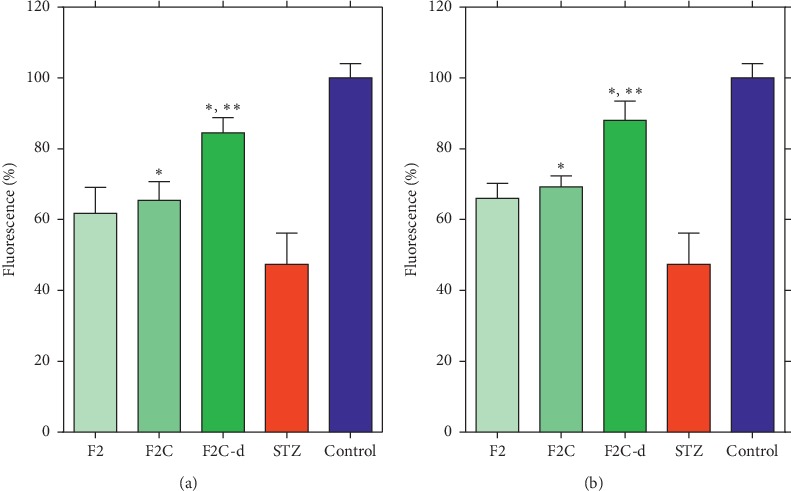
The effect of coadministration (a) and pretreatment (b) of F2, F2C, and F2C-d on STZ-induced (48 *μ*g/*μ*L) mitochondrial membrane potential (MMP) collapse. After 24 h of treatment, the MMP was measured through rhodamine 123 fluorescence. Data are expressed as the mean ± SD of three separate experiments. Significance was calculated by ANOVA. ^*∗*^Significantly different from the STZ group; ^*∗∗*^significantly different from F2 at *p* ≤ 0.05.

Mitochondria membrane potential (MMP) is commonly measured using the lipophilic cation, rhodamine 123, which readily passes the mitochondrial membrane and accumulated in the mitochondria. MMP is tightly linked to the mitochondrial function and a decrease in the MMP could result from apoptosis induced by STZ. As assessed by rhodamine 123 fluorescence, STZ decreased the MMP by up to 50% of the control group in *β*-TC_3_ cell line (Figure 5). Pretreatment or co-treatment of cells with the active fractions (F2, F2C, and F2C-d), however, significantly increases the intracellular fluorescence (*p* < 0.005) of *β*-TC_3_ cells. As with the cytoprotective effect, there was no significant difference between pretreatment and coadministration (Figure 5). The MMP result demonstrated that there was no significant difference between F2 and F2C fractions, while F2C-d was the most active.

### 3.4. Effect of F2, F2C, and F2C-d Fractions on Glucose Consumption in HepG2 Cells

**Figure 6:**
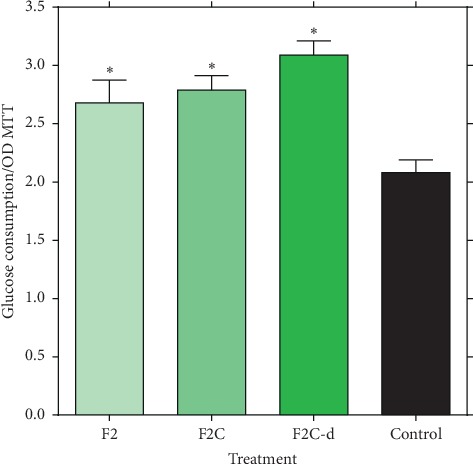
The effect of fractions of F2, F2C, and F2C-d on glucose consumption in HepG2 cells. Statistical significance verses the control group was calculated by ANOVA (^*∗*^*p* ≤ 0.05).

Under high-glucose or hyperglycemic (11.1 mM glucose) condition, the 0.5 mg/mL of the active extracts/fractions consumption in hepatic HepG2 cells was assessed. As shown in Figure 6, treatment with the active fractions for 24 h showed the glucose-lowering effect of F2, F2C, and F2C-d. Once more, the higher activity of fraction F2C-d was evident while there was no significant difference between F2 and F2C. Since glucose consumption was normalized by viable cells (Figure 6), the observed increase in glucose consumption was not due to an increment of cell number.

### 3.5. Preliminary NMR Analysis of F2C-d

**Figure 7:**
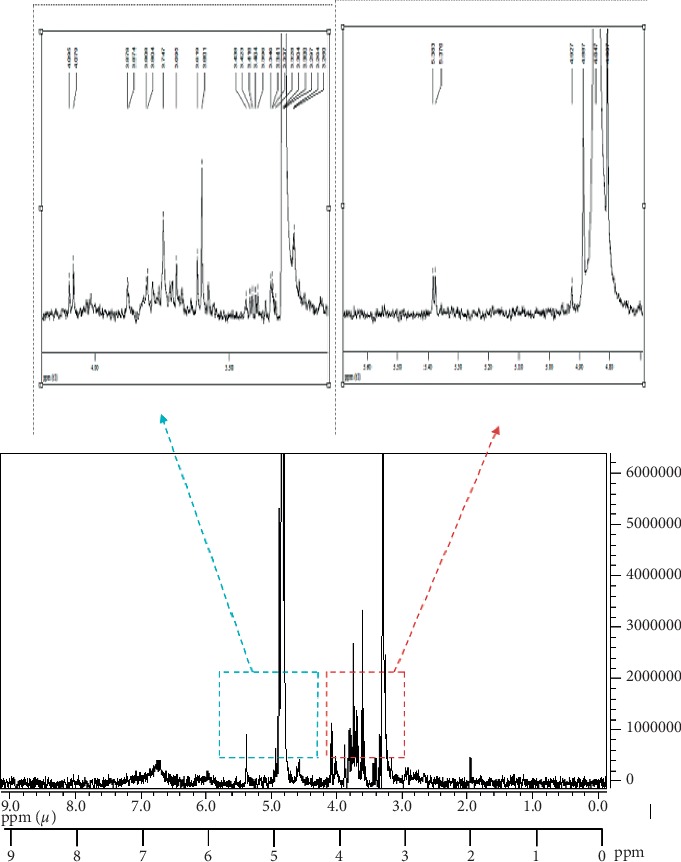
The ^1^H NMR (500 MHz) spectrum of F2C-d in CD_3_OD : D_2_O.

Preliminary ^1^H NMR analysis of the most active fraction (F2C-d) showed the classical sugar region resonances (3 to 5 ppm) suggesting possible complex polysaccharide or the oligosaccharide nature of the active compound (Figure 7). The small amount of the sample obtained in our experiment did not permit more studies on detailed structural elucidation of the active principle.

## 4. Discussion

The various parts of *P. farcta* have been shown to display a range of pharmacological activities. For example, the fruits have been shown to display antidiabetic effect in STZ-induced diabetic rats [17]. The fruits are also shown to increase the high-density lipoprotein (HDL) cholesterol while decreasing low-density lipoprotein (LDL) cholesterol in ostriches suggesting its potential lipid-lowering effect although improvement of lipid dysregulation under diabetic conditions was not demonstrated for the fruits extract in animal models [15]. On the other hand, antioxidant effect in the diabetes model was illustrated [13]: an effect that was linked to the presence of antioxidant polyphenolic compounds such as quercetin in the plant [16]. The antioxidant activity as a mechanism of biological action for the plant was also suggested [19].

In the present study, we used an efficient in vitro model using two of the most diabetes-relevant targets: hepatocytes and pancreatic *β*-cells. To date, the most widely used diabetes model is based on induction of pancreatic *β*-cells loss by injecting the antibacterial, antitumor, and carcinogenic antibiotic, STZ [20]. Accumulated in *β*-cells via uptake through the glucose transporter 2 (GLUT2), STZ, can induce diabetes through direct cytotoxic effect against *β*-cells [21]. As a mechanism of this cytotoxic effect, methylation of DNA through the activation of the poly-ADP-ribose synthetase and consequently, NAD^+^ depletion, nitric oxide production, free radical generation, and modulation of the NF-*κ*B-based cell signal transduction pathway have been suggested [22]. The induction of cell death in pancreatic *β*-cell by STZ also involve the generation of intracellular reactive oxygen species (ROS) [23]. Hence, the direct protective effect of natural products in *β*-cells is regarded as a valid antidiabetic mechanism of action.

Natural products can induce antidiabetic effect through diverse mechanisms including suppression of glucose availability from the intestine or glucose production in the liver, enhancing the glucose uptake by tissues, increasing insulin secretion from *β*-cells, and increasing pancreatic tissue regeneration [24]. In the present study, we first demonstrated that concentrations up to 0.5 mg/mL of the tested water extracts and/or fractions obtained from the roots of *P. farcta* do not induce cytotoxic effect in *β*-TC3 cell in vitro. When these nontoxic concentrations were coadministered or added 6 h before STZ, cytoprotective effects were demonstrated. We then followed a bioassay-guided fractionation procedure and successfully isolated the active principle(s) that appear to be polysaccharide/sugar nature on the basis of preliminary NMR analysis. The overall bioassay-guided fractionation procedure is depicted in Scheme 1. Hence, our study appears to lay down the foundation for the isolation of the polysaccharide-based active principle in the future studies.

The mitochondria being the center of ROS production and apoptosis signaling, we measured the potential effect of the active fractions on MMP. Our data have shown that the active fractions could modulate the MMP. As evidenced from the rhodamine 123 fluorescence study, amelioration of the STZ-effect on mitochondrial changes suggests the antidiabetic potential of the roots of *P. farcta.*

The other most important diabetes target is the liver which is central to glucose metabolism and insulin resistance. Various antidiabetic agents of clinical relevance including metformin increase the glycogen synthesis and/or suppress glucose production in the liver [25]. Hence, our bioassay also included glucose uptake and/or utilization in hepatocytes (HepG2 cells) in vitro. Interestingly, the fractions and the active principle identified in pancreatic *β*-cell protection assay also displayed activity, i.e., it increased glucose consumption in these cells. This could be a result of either increased glucose uptake or utilization, but the detailed mechanism of action remains to be elucidated.

Numerous studies on plant-based antidiabetic agents have shown the identification of polysaccharides as active principle. This include inhibition of *β*-cell apoptosis and enhancement of *β*-cell viability/numbers by polysaccharides extracted from mulberry leaf [26], *Ganoderma atrum* [27], *Ganoderma lucidum* fruiting bodies [28], and pumpkin fruits [29]. Other examples include the antidiabetic effects demonstrated for the polysaccharides from *Salvia miltiorrhiza* [30], fruit body of *Grifola frondosa* [31, 32], *Catathelasma ventricosum* [32], and roots of *Ophiopogon japonica* [33]. In line with our observation in the present study, the antidiabetic potential of polysaccharides by inhibiting glucose uptake has been shown previously [34]. Further studies are however required to establish the exact antidiabetic mechanism of the roots of *P. farcta* or the active sugar component. Further studies are also necessary to isolate and elucidate the structures of the saccharide(s) presented in F2C-d.

## 5. Conclusion

The study demonstrated that the water extract of *P. farcta* could protect *β*-TC3 cells from STZ-induced cytotoxicity in vitro. Bioassay-guided fractionation of the water extract resulted in the isolation of a carbohydrate/sugar fractions as active principle. The active principle was also a better protective agent against the STZ-induced MMP change and cytotoxicity in these cells. Furthermore, augmentation of glucose consumption in HEPG2 hepatocyte cells was more prevalent for the isolated compound/fraction than crude fractions. Further study is thus well merited to undertake large-scale isolation of the active components for further mechanistic and potential antidiabetic assessments.

## Figures and Tables

**Table 1:** Effect of various fractions of the root extract of *P. farcta* on the viability of *β*-TC_3_ cells^*∗*^.

Fractions	Concentrations (mg/mL)			
	0.01	0.05	0.1	0.5
F1	96.0 ± 8	98.0 ± 7.0	100.0 ± 7.0	108.0 ± 9.0
F2	103.0 ± 8.7	105.0 ± 10	107.0 ± 6.0	110.0 ± 8.0
F3	92 ± 9	93.0 ± 9.7	97.0 ± 8.0	107.0 ± 4.0
F4	104.0 ± 4.75	107.3 ± 4.6	108.1 ± 5.2	109.0 ± 6.1
F5	94.7 ± 8.18	96.2 ± 5.6	98.32 ± 5.38	103.2 ± 6.1
F6	95.2 ± 3.8	104.2 ± 4.14	108 ± 4.1	110.0 ± 6
F7	105.53 ± 8	108.6 ± 6.35	110.3 ± 6.98	114.8 ± 7.2
F2A	89.9 ± 7.34	96.9 ± 15	105 ± 4.14	114.2 ± 7.8
F2B	87.9 ± 8.3	90.0 ± 10.7	96.94 ± 7.82	98.07 ± 6.6
F2C	91.0 ± 8.7	103.0 ± 8.0	111.7 ± 6.0	115 ± 8.0
F2D	96.3 ± 3.2	98.07 ± 4.5	95 ± 7.4	112.56 ± 8.5
F2C-a	88.8 ± 3.7	88.23 ± 6.9	86.8 ± 6.5	84.8 ± 4.6
F2C-b	84.31 ± 7.1	85.27 ± 5.2	84.5 ± 5.5	87.7 ± 2.7
F2C-c	92.1 ± 7.5	96.4 ± 7.0	88.7 ± 12.0	94.7 ± 9.3
F2C-d	93.9 ± 6.5	97.0 ± 8.28	99.0 ± 10.23	108.5 ± 14.0

^*∗*^Cell viability was determined by the MTT assay after 24 h of treatment with the various fractions. Data are expressed as the mean ± SD of three separate experiments and were not significantly different from the untreated control group at the level of *p*=0.05.

## Data Availability

The data generated and/or analyzed during the current study are available from the corresponding authors on reasonable request.
